# Crystal structure of (*E*)-dodec-2-enoic acid

**DOI:** 10.1107/S2056989015011937

**Published:** 2015-06-30

**Authors:** Marcel Sonneck, Tim Peppel, Anke Spannenberg, Sebastian Wohlrab

**Affiliations:** a Leibniz-Institut für Katalyse e. V. an der Universität Rostock, Albert-Einstein-Str. 29a, 18059 Rostock, Germany

**Keywords:** crystal structure, hydrogen bonding, dimer, unsaturated carb­oxy­lic acid, fatty acid

## Abstract

The crystal structure of (*E*)-dodec-2-enoic acid, C_12_H_22_O_2_, an α,β-unsaturated carb­oxy­lic acid with a melting point (295 K) near room temperature, is characterized by carb­oxy­lic acid inversion dimers linked by pairs of O—H⋯O hydrogen bonds. The carb­oxy­lic acid group and the following three carbon atoms of the chain of the (*E*)-dodec-2-enoic acid mol­ecule lie almost in one plane (r.m.s. deviation for the four C atoms and two O atoms = 0.012 Å), whereas the remaining carbon atoms of the hydro­carbon chain adopt a nearly fully staggered conformation [moduli of torsion angles vary from 174.01 (13) to 179.97 (13)°].

## Related literature   

For the synthesis of unsaturated α,β-carb­oxy­lic acids including the title compound by adapted routes established by Knoevenagel (1898 ▸) and Doebner (1902 ▸), see: Shabtai *et al.* (1981 ▸). For crystal structure determinations of related α,β-unsaturated carb­oxy­lic acids, see, for acrylic acid: Higgs *et al.* (1963 ▸), Chatani *et al.* (1963 ▸), Boese *et al.* (1999 ▸), or Oswald *et al.* (2011 ▸); see, for crotonic acid: Shimizu *et al.* (1974 ▸); see, for (*E*)-pent-2-enoic acid: Peppel *et al.* (2015*a* ▸); see, for (*E*)-hex-2-enoic acid: Peppel *et al.* (2015*b* ▸); see, for (*E*)-undecen-2-enoic acid: Sonneck *et al.* (2015 ▸). For structures of co-crystals containing (*E*)-hex-2-enoic acid, see: Aakeröy *et al.* (2003 ▸), or Stanton & Bak (2008 ▸).
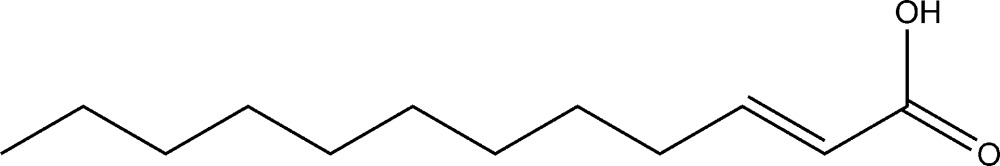


## Experimental   

### Crystal data  

C_12_H_22_O_2_
*M* *_r_* = 198.29
Triclinic, 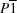

*a* = 4.6475 (2) Å
*b* = 5.4169 (2) Å
*c* = 24.7041 (10) Å
α = 91.547 (2)°
β = 91.788 (2)°
γ = 102.3158 (19)°
*V* = 606.96 (4) Å^3^
*Z* = 2
Cu *K*α radiation
μ = 0.56 mm^−1^
*T* = 150 K
0.44 × 0.30 × 0.12 mm

### Data collection  

Bruker APEXII CCD diffractometer
Absorption correction: multi-scan (*SADABS*; Bruker, 2014 ▸) *T*_min_ = 0.80, *T*_max_ = 0.94
12048 measured reflections
2117 independent reflections
1964 reflections with *I* > 2σ(*I*)
*R*_int_ = 0.026

### Refinement  

*R*[*F* ^2^ > 2σ(*F* ^2^)] = 0.046
*wR*(*F* ^2^) = 0.131
*S* = 1.13
2117 reflections
129 parameters
H-atom parameters constrained
Δρ_max_ = 0.29 e Å^−3^
Δρ_min_ = −0.23 e Å^−3^

### 

Data collection: *APEX2* (Bruker, 2014 ▸); cell refinement: *SAINT* (Bruker, 2013 ▸); data reduction: *SAINT*; program(s) used to solve structure: *SHELXS97* (Sheldrick, 2008 ▸); program(s) used to refine structure: *SHELXL2014*/7 (Sheldrick, 2015 ▸); molecular graphics: *SHELXL2014*/7; software used to prepare material for publication: *SHELXL2014*/7.

## Supplementary Material

Crystal structure: contains datablock(s) I, New_Global_Publ_Block. DOI: 10.1107/S2056989015011937/hb7452sup1.cif

Structure factors: contains datablock(s) I. DOI: 10.1107/S2056989015011937/hb7452Isup2.hkl

Supporting information file. DOI: 10.1107/S2056989015011937/hb7452Isup3.cml

**Fig. 1.**
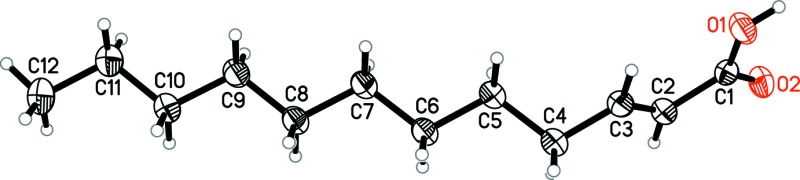
Molecular structure of the title compound with atom labelling and displacement ellipsoids drawn at 50% probability level.

. DOI: 10.1107/S2056989015011937/hb7452fig1.tif
Mol­ecular structure of the title compound with atom labelling and displacement ellipsoids drawn at 50% probability level.

**Fig. 2.**
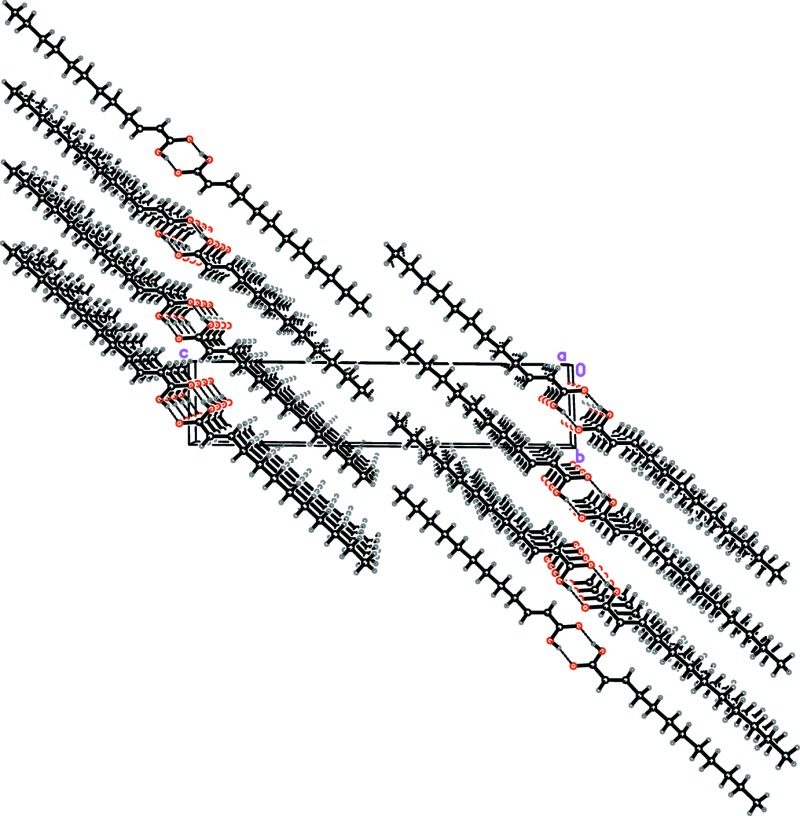
Packing diagram showing intermolecular hydrogen bonding.

. DOI: 10.1107/S2056989015011937/hb7452fig2.tif
Packing diagram showing inter­molecular hydrogen bonding.

CCDC reference: 1407997

Additional supporting information:  crystallographic information; 3D view; checkCIF report

## Figures and Tables

**Table 1:** Hydrogen-bond geometry (, )

*D*H*A*	*D*H	H*A*	*D* *A*	*D*H*A*
O1H1O2^i^	0.84	1.80	2.6319(15)	170

Symmetry code: (i) 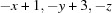.

## supplementary crystallographic information

### S1. Synthesis and crystallization

Malonic acid (25.0 g, 240.2 mmol, 1.0 eq) is dissolved in dry pyridine (38.0 g, 480.5 mmol, 2.0 eq) at room temperature in a three-necked flask equipped with a magnetic stir bar and a reflux condenser under a mild flow of argon. Decanal (37.5 g, 240.2 mmol, 1.0 eq) is then added in one portion and the resulting clear solution is further stirred for 72 h at room temperature under argon. Afterwards, the resulting light yellow to orange solution is brought to an acidic pH value by adding phospho­ric acid at 0 °C (42.5wt. %, 138.5 g, 600.6 mmol, 2.5 eq). The resulting two layers are extracted three times with 150 mL portions of ethyl acetate and reduced to a volume of ca. 150 mL *in vacuo*. To remove impurities from aldol condensation the raw acid is converted into the corresponding sodium salt by addition of an aqueous solution of sodium carbonate (20.4 g, 192.2 mmol, 0.8 eq in 200 mL). After stirring for 30 minutes the water phase is separated and extracted three times with 150 mL portions of ethyl acetate. The water phase is then acidified with concentrated hydro­chloric acid (37.0wt. %, 35.5 g, 360.4 mmol, 1.5 eq), the organic phase is separated and the water phase is again extracted three times with 150 mL portions of ethyl acetate. The combined organic phases are dried over Na_2_SO_4_ and evaporated to dryness under diminished pressure. The resulting raw product is further purified by distillation *in vacuo* yielding the product in purity >99% (GC). M. p. 22 °C. ^1^H NMR (400 MHz, CDCl_3_): δ = 12.15 (br s, 1H, OH); 7.09 (dt, ^3^*J* = 15.6 Hz, ^3^*J* = 7.0 Hz, 1H, -CH-); 5.82 (dt, ^3^*J* = 15.6 Hz, ^4^*J* = 1.6 Hz, 1H, -CH-); 2.26-2.19 (m, 2H, -CH_2_-); 1.50-1.42 (m, 2H, -CH_2_-); 1.32-1.24 (m, 12H, 6x -CH_2_-); 0.90-0.86 (m, 3H, -CH_3_-). ^13^C NMR (100 MHz, CDCl_3_): δ = 172.53 (CO); 152.68 (CH); 120.76 (CH); 32.47 (CH_2_); 32.02 (CH_2_); 29.61 (CH_2_), 29.52 (CH_2_), 29.43 (CH_2_); 29.29 (CH_2_); 28.02 (CH_2_); 22.81 (CH_2_); 14.23 (CH_3_). MS (EI, 70eV): *m/z* = 198 (M^+^, 0), 99 (16), 98 (12), 97 (17), 96 (14), 95 (11), 86 (17), 84 (18), 83 (16), 82 (12), 81 (20), 73 (34), 71 (13), 70 (15), 69 (21), 68 (18), 67 (20), 57 (29), 56 (22), 55 (47), 54 (11), 53 (20), 45 (14), 43 (70), 42 (19), 41 (100), 40 (14), 39 (52), 29 (58). HRMS (ESI-TOF/MS): calculated for C_12_H_22_O_2_ ([M—H]^-^) 197.1547, found 197.15481. Elemental analysis for C_12_H_22_O_2_ % (calc.): C 72.53 (72.68); H 11.24 (11.18). Suitable single crystals were grown by slow evaporation of an ethano­lic solution at -30 °C over one week.

### S2. Refinement

H atoms were placed in idealized positions with d(C—H) = 0.95 Å (CH), 0.99 Å (CH_2_), 0.98 Å (CH_3_) and refined using a riding model with *U*_iso_(H) fixed at 1.2 *U*_eq_(C) for CH and CH_2_ and 1.5 *U*_eq_(C) for CH_3_. The carb­oxy­lic acid group was assigned by examining the C—O distances and H1 was placed using the AFIX 147 instruction (d(O—H) = 0.84 Å, *U*_iso_(H) fixed at 1.5 *U*_eq_(O)).

### Crystal data

**Table:**

C_12_H_22_O_2_	*Z* = 2
*M**_r_* = 198.29	*F*(000) = 220
Triclinic, *P*1	*D*_x_ = 1.085 Mg m^−^^3^
*a* = 4.6475 (2) Å	Cu *K*α radiation, λ = 1.54178 Å
*b* = 5.4169 (2) Å	Cell parameters from 6666 reflections
*c* = 24.7041 (10) Å	θ = 3.6–66.9°
α = 91.547 (2)°	µ = 0.56 mm^−^^1^
β = 91.788 (2)°	*T* = 150 K
γ = 102.3158 (19)°	Plate, colourless
*V* = 606.96 (4) Å^3^	0.44 × 0.30 × 0.12 mm

### Data collection

**Table:**

Bruker APEXII CCD diffractometer	2117 independent reflections
Radiation source: microfocus	1964 reflections with *I* > 2σ(*I*)
Multilayer monochromator	*R*_int_ = 0.026
Detector resolution: 8.3333 pixels mm^-1^	θ_max_ = 66.0°, θ_min_ = 3.6°
φ and ω scans	*h* = −5→5
Absorption correction: multi-scan (*SADABS*; Bruker, 2014)	*k* = −6→6
*T*_min_ = 0.80, *T*_max_ = 0.94	*l* = −29→29
12048 measured reflections	

### Refinement

**Table:**

Refinement on *F*^2^	0 restraints
Least-squares matrix: full	Hydrogen site location: inferred from neighbouring sites
*R*[*F*^2^ > 2σ(*F*^2^)] = 0.046	H-atom parameters constrained
*wR*(*F*^2^) = 0.131	*w* = 1/[σ^2^(*F*_o_^2^) + (0.0529*P*)^2^ + 0.282*P*] where *P* = (*F*_o_^2^ + 2*F*_c_^2^)/3
*S* = 1.13	(Δ/σ)_max_ < 0.001
2117 reflections	Δρ_max_ = 0.29 e Å^−^^3^
129 parameters	Δρ_min_ = −0.23 e Å^−^^3^

### Special details

**Table:**

Geometry. All e.s.d.'s (except the e.s.d. in the dihedral angle between two l.s. planes) are estimated using the full covariance matrix. The cell e.s.d.'s are taken into account individually in the estimation of e.s.d.'s in distances, angles and torsion angles; correlations between e.s.d.'s in cell parameters are only used when they are defined by crystal symmetry. An approximate (isotropic) treatment of cell e.s.d.'s is used for estimating e.s.d.'s involving l.s. planes.

### Fractional atomic coordinates and isotropic or equivalent isotropic displacement parameters (Å^2^)

**Table:**

	*x*	*y*	*z*	*U*_iso_*/*U*_eq_	
C1	0.7187 (3)	1.2963 (3)	0.03952 (6)	0.0277 (4)	
C2	0.8819 (3)	1.1356 (3)	0.06965 (6)	0.0314 (4)	
H2	0.9763	1.0250	0.0497	0.038*	
C3	0.9029 (3)	1.1384 (3)	0.12290 (6)	0.0306 (4)	
H3	0.8092	1.2517	0.1421	0.037*	
C4	1.0622 (4)	0.9777 (3)	0.15566 (7)	0.0332 (4)	
H4A	1.2230	1.0881	0.1778	0.040*	
H4B	1.1523	0.8714	0.1309	0.040*	
C5	0.8573 (3)	0.8076 (3)	0.19299 (6)	0.0303 (4)	
H5A	0.7510	0.9129	0.2148	0.036*	
H5B	0.7090	0.6858	0.1706	0.036*	
C6	1.0184 (3)	0.6615 (3)	0.23115 (6)	0.0304 (4)	
H6A	1.1303	0.5606	0.2094	0.036*	
H6B	1.1618	0.7832	0.2545	0.036*	
C7	0.8121 (4)	0.4855 (3)	0.26692 (6)	0.0311 (4)	
H7A	0.6701	0.3631	0.2435	0.037*	
H7B	0.6985	0.5865	0.2882	0.037*	
C8	0.9696 (4)	0.3400 (3)	0.30579 (6)	0.0313 (4)	
H8A	1.0830	0.2386	0.2846	0.038*	
H8B	1.1115	0.4622	0.3293	0.038*	
C9	0.7607 (4)	0.1648 (3)	0.34138 (7)	0.0333 (4)	
H9A	0.6186	0.0429	0.3178	0.040*	
H9B	0.6474	0.2664	0.3626	0.040*	
C10	0.9160 (4)	0.0181 (3)	0.38039 (7)	0.0341 (4)	
H10A	1.0257	−0.0866	0.3592	0.041*	
H10B	1.0612	0.1398	0.4035	0.041*	
C11	0.7074 (4)	−0.1519 (4)	0.41657 (8)	0.0432 (4)	
H11A	0.5556	−0.2675	0.3936	0.052*	
H11B	0.6057	−0.0462	0.4393	0.052*	
C12	0.8622 (5)	−0.3083 (4)	0.45332 (8)	0.0495 (5)	
H12A	0.9605	−0.4159	0.4311	0.074*	
H12B	0.7169	−0.4145	0.4755	0.074*	
H12C	1.0089	−0.1952	0.4770	0.074*	
O1	0.5999 (3)	1.4499 (2)	0.06736 (5)	0.0374 (3)	
H1	0.5041	1.5251	0.0464	0.056*	
O2	0.7029 (3)	1.2789 (2)	−0.01126 (4)	0.0363 (3)	

### Atomic displacement parameters (Å^2^)

**Table:**

	*U*^11^	*U*^22^	*U*^33^	*U*^12^	*U*^13^	*U*^23^
C1	0.0262 (7)	0.0246 (8)	0.0319 (8)	0.0035 (6)	0.0022 (6)	0.0044 (6)
C2	0.0306 (8)	0.0301 (8)	0.0352 (9)	0.0100 (7)	0.0022 (6)	0.0046 (6)
C3	0.0274 (8)	0.0291 (8)	0.0358 (9)	0.0071 (6)	0.0010 (6)	0.0042 (6)
C4	0.0309 (8)	0.0359 (9)	0.0347 (9)	0.0110 (7)	−0.0009 (6)	0.0067 (7)
C5	0.0300 (8)	0.0306 (8)	0.0318 (8)	0.0104 (7)	−0.0016 (6)	0.0032 (6)
C6	0.0294 (8)	0.0295 (8)	0.0334 (8)	0.0095 (7)	−0.0032 (6)	0.0030 (6)
C7	0.0311 (8)	0.0301 (8)	0.0335 (8)	0.0102 (7)	−0.0021 (6)	0.0033 (6)
C8	0.0311 (8)	0.0301 (8)	0.0342 (8)	0.0098 (7)	−0.0023 (6)	0.0040 (7)
C9	0.0327 (8)	0.0335 (9)	0.0347 (9)	0.0096 (7)	−0.0019 (7)	0.0051 (7)
C10	0.0351 (9)	0.0342 (9)	0.0347 (9)	0.0109 (7)	−0.0010 (7)	0.0061 (7)
C11	0.0415 (10)	0.0459 (11)	0.0433 (10)	0.0103 (8)	0.0025 (8)	0.0147 (8)
C12	0.0554 (12)	0.0499 (11)	0.0450 (11)	0.0127 (9)	0.0035 (9)	0.0192 (9)
O1	0.0442 (7)	0.0364 (7)	0.0367 (6)	0.0193 (5)	0.0013 (5)	0.0060 (5)
O2	0.0453 (7)	0.0357 (7)	0.0307 (6)	0.0143 (5)	−0.0012 (5)	0.0053 (5)

### Geometric parameters (Å, º)

**Table:**

C1—O2	1.2543 (19)	C7—H7B	0.9900
C1—O1	1.2881 (19)	C8—C9	1.524 (2)
C1—C2	1.473 (2)	C8—H8A	0.9900
C2—C3	1.316 (2)	C8—H8B	0.9900
C2—H2	0.9500	C9—C10	1.524 (2)
C3—C4	1.496 (2)	C9—H9A	0.9900
C3—H3	0.9500	C9—H9B	0.9900
C4—C5	1.527 (2)	C10—C11	1.518 (2)
C4—H4A	0.9900	C10—H10A	0.9900
C4—H4B	0.9900	C10—H10B	0.9900
C5—C6	1.525 (2)	C11—C12	1.523 (2)
C5—H5A	0.9900	C11—H11A	0.9900
C5—H5B	0.9900	C11—H11B	0.9900
C6—C7	1.522 (2)	C12—H12A	0.9800
C6—H6A	0.9900	C12—H12B	0.9800
C6—H6B	0.9900	C12—H12C	0.9800
C7—C8	1.524 (2)	O1—H1	0.8400
C7—H7A	0.9900		
			
O2—C1—O1	123.37 (14)	H7A—C7—H7B	107.7
O2—C1—C2	119.24 (14)	C9—C8—C7	113.34 (13)
O1—C1—C2	117.38 (13)	C9—C8—H8A	108.9
C3—C2—C1	122.91 (15)	C7—C8—H8A	108.9
C3—C2—H2	118.5	C9—C8—H8B	108.9
C1—C2—H2	118.5	C7—C8—H8B	108.9
C2—C3—C4	125.30 (15)	H8A—C8—H8B	107.7
C2—C3—H3	117.4	C8—C9—C10	113.76 (13)
C4—C3—H3	117.4	C8—C9—H9A	108.8
C3—C4—C5	112.03 (13)	C10—C9—H9A	108.8
C3—C4—H4A	109.2	C8—C9—H9B	108.8
C5—C4—H4A	109.2	C10—C9—H9B	108.8
C3—C4—H4B	109.2	H9A—C9—H9B	107.7
C5—C4—H4B	109.2	C11—C10—C9	113.50 (14)
H4A—C4—H4B	107.9	C11—C10—H10A	108.9
C6—C5—C4	113.32 (13)	C9—C10—H10A	108.9
C6—C5—H5A	108.9	C11—C10—H10B	108.9
C4—C5—H5A	108.9	C9—C10—H10B	108.9
C6—C5—H5B	108.9	H10A—C10—H10B	107.7
C4—C5—H5B	108.9	C10—C11—C12	113.18 (15)
H5A—C5—H5B	107.7	C10—C11—H11A	108.9
C7—C6—C5	113.10 (13)	C12—C11—H11A	108.9
C7—C6—H6A	109.0	C10—C11—H11B	108.9
C5—C6—H6A	109.0	C12—C11—H11B	108.9
C7—C6—H6B	109.0	H11A—C11—H11B	107.8
C5—C6—H6B	109.0	C11—C12—H12A	109.5
H6A—C6—H6B	107.8	C11—C12—H12B	109.5
C6—C7—C8	113.85 (13)	H12A—C12—H12B	109.5
C6—C7—H7A	108.8	C11—C12—H12C	109.5
C8—C7—H7A	108.8	H12A—C12—H12C	109.5
C6—C7—H7B	108.8	H12B—C12—H12C	109.5
C8—C7—H7B	108.8	C1—O1—H1	109.5
			
O2—C1—C2—C3	−178.37 (15)	C5—C6—C7—C8	−179.36 (13)
O1—C1—C2—C3	1.9 (2)	C6—C7—C8—C9	179.97 (13)
C1—C2—C3—C4	179.24 (14)	C7—C8—C9—C10	179.96 (13)
C2—C3—C4—C5	−120.08 (17)	C8—C9—C10—C11	178.76 (14)
C3—C4—C5—C6	−174.01 (13)	C9—C10—C11—C12	176.92 (15)
C4—C5—C6—C7	−178.05 (13)		

### Hydrogen-bond geometry (Å, º)

**Table:**

*D*—H···*A*	*D*—H	H···*A*	*D*···*A*	*D*—H···*A*
O1—H1···O2^i^	0.84	1.80	2.6319 (15)	170

Symmetry code: (i) −*x*+1, −*y*+3, −*z*.
