# Supplementary figures and images for: Crystal structure of (E)-dodec-2-enoic acid
Source: Acta Crystallogr E Crystallogr Commun. 2015 Jun 30;71(Pt 7):o528–9. doi: 10.1107/S2056989015011937 (PMC4518913; doi:10.1107/S2056989015011937)

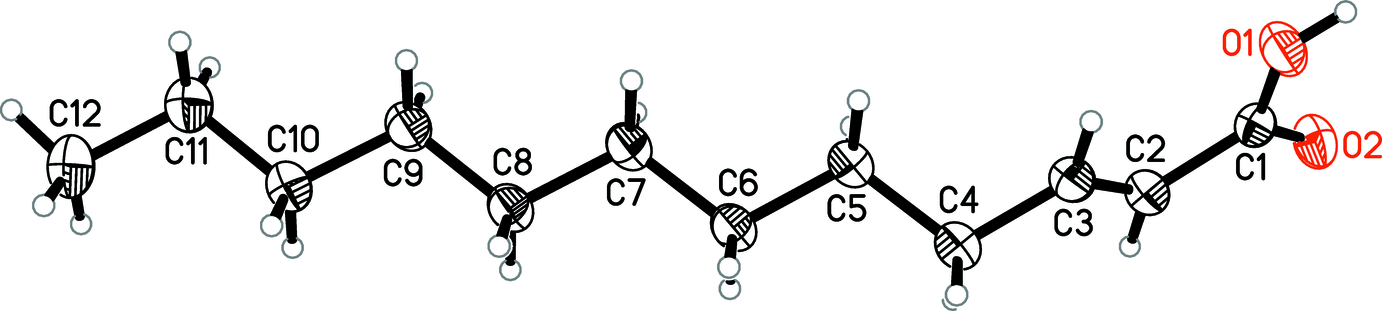

Supplement: Supplementary file 4 [file e-71-0o528-fig1.tif]

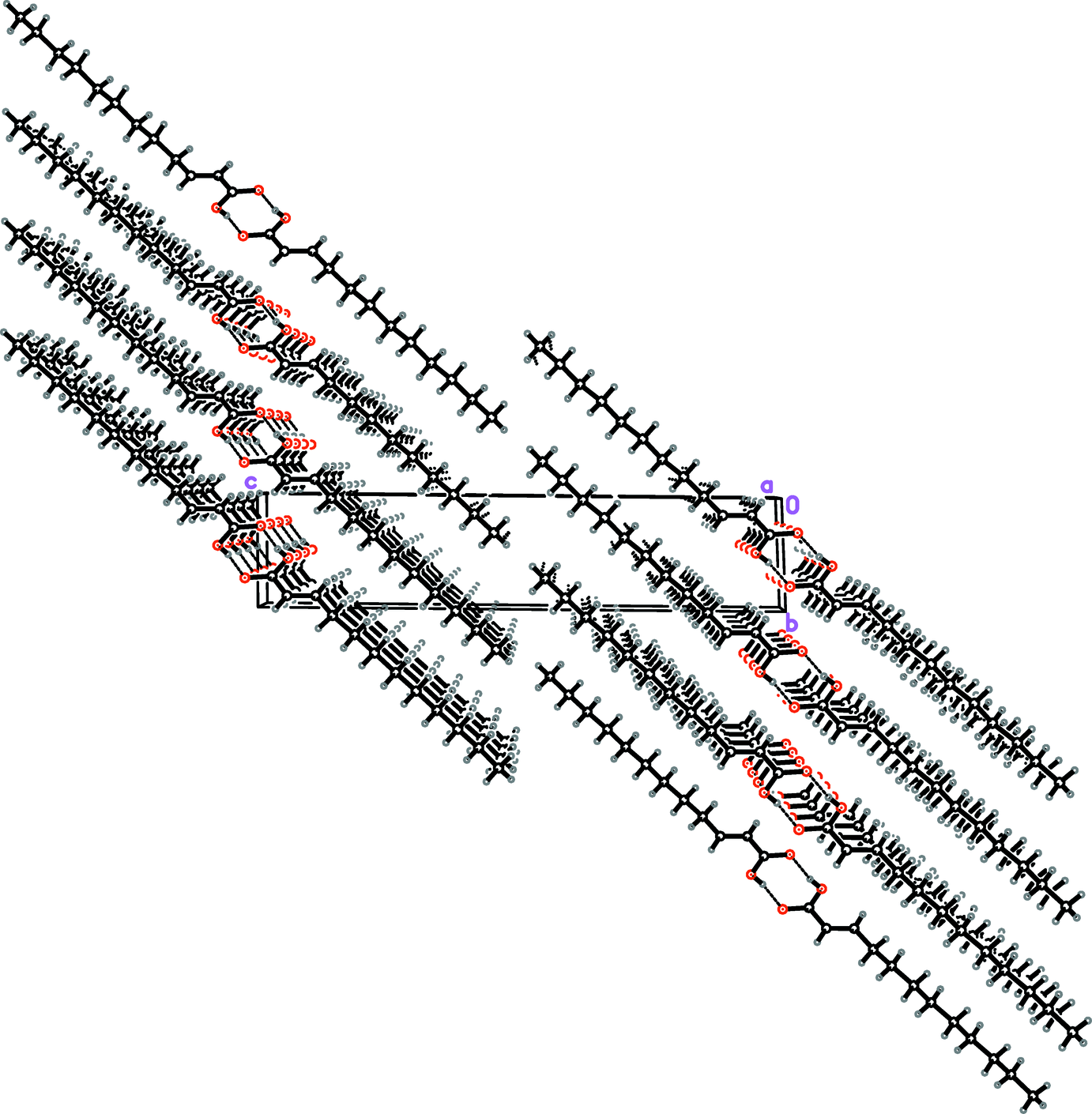

Supplement: Supplementary file 5 [file e-71-0o528-fig2.tif]
